# The application of the theory of planned behavior to nutritional behaviors related to cardiovascular disease among the women

**DOI:** 10.1186/s12872-021-02399-3

**Published:** 2021-12-07

**Authors:** Ali Khani Jeihooni, Hanieh Jormand, Negin Saadat, Mahmood Hatami, Rosliza Abdul Manaf, Pooyan Afzali Harsini

**Affiliations:** 1 Nutrition Research Center, Department of Public Health, School of Health, Shiraz University of Medical Sciences, Shiraz, Iran; 2 Department of Public Health, School of Health and Autism Spectrum Disorders Research Center, Hamedan University of Medical Sciences, Hamedan, Iran; 3 Departement of Public Health, School of Health, Fasa University of Medical Sciences, Fasa, Iran; 4 Department of Nursing, School of Nursing, Fasa University of Medical Sciences, Fasa, Iran; 5 Department of Community Health, Faculty of Medicine & Health Sciences, Universiti Putra, Selangor, Malaysia; 6 Department of Public Health, School of Health, Kermanshah University of Medical Sciences, Kermanshah, Iran; 7 Nutrition Research Center, Department of Public Health, School of Health, Shiraz University of Medical Sciences, Shiraz, Iran

**Keywords:** Cardiovascular disease, Nutritional behaviors, Theory of planned behavior, Educational intervention

## Abstract

**Background:**

Nutritional factors have been identified as preventable risk factors for cardiovascular disease; this study aimed to investigate the application of the Theory of Planned Behavior (TPB) in nutritional behaviors related to cardiovascular diseases among the women in Fasa city, Fars province, Iran.

**Methods:**

The study was conducted in two stages. First, the factors affecting nutritional behaviors associated with cardiovascular disease on 350 women who were referred to Fasa urban health centers were determined based on the TPB. In the second stage, based on the results of a cross-sectional study, quasi-expeimental study was performed on 200 women covered by Fasa health centers. The questionnaire used for the study was a questionnaire based on TPB. The questionnaire was completed by the experimental and control groups before and three months after the intervention. Data were analyzed by SPSS software using logistic regression, paired t-test, independent sample t-test, and chi-square test. The level of significance is considered 0.05.

**Result:**

The constructs of attitude, subjective norms, and perceived behavioral control (PBC) were predictors of nutritional behaviors associated with cardiovascular disease in women. The constructs predicted 41.6% of the behavior. The results showed that mean scores of attitude, subjective norms, PBC, intention, nutritional performance related to the cardiovascular disease before intervention were, respectively, 24.32, 14.20, 18.10, 13.37 and 16.28, and after the intervention, were, respectively, 42.32, 25.40, 33.72, 30.13 and 41.38. All the constructs except the attitude in the intervention group were significantly higher (*p* < 0.001) than the control group.

**Conclusion:**

The results of the present study showed that the educational intervention based on the TPB would be consider an effective educational and promotinal strategy for the nutritional behaviors to prevent cardiovascular disease in women. Considering the role of mothers in providing family food baskets and the effect of their nutritional behaviors on family members, the education of this group can promote healthy eating behaviors in the community and family**.**

## Background

A cardiovascular disease is a group of non-communicable diseases that is the main cause of death worldwide with an estimated 17.5 million deaths annually [1, 2]. Cardiovascular disease (CVD) would be the cause of more than 23 million (about 30.5%) deaths by 2030 worldwide [1, 3, 4]. These diseases can lead to hospitalization, disability, and a decline in quality of life [5]. Also, the economic burden of cardiovascular disease is high, so that in the United States in 2010, approximately $315 million was spent on this disease and is estimated to rise to $818 billion by 2030 [6, 7].

Cardiovascular disease is generally thought to affect men more than women, while it is not true [8]. According to the statistics, when a heart attack occurs, women die twice as much as men in the first week, and the death rate in women, 1 year after a heart attack, is 13% higher than men [9]. Tobacco use, inactivity, hypertension, hyperlipidemia, diabetes, obesity, and overweight, stress, contraceptives, and alcohol use are risk factors for cardiovascular disease [10–12]. CVD can be prevented by modifying risk factors and increasing knowledge [13, 14]. To adopt healthy behaviors, people must be aware of the disease and, considering themselves susceptible to the disease, believe that they can prevent or cure the disease [15]. Nutritional factors have been identified as preventable risk factors for cardiovascular disease [16]. Most of the major risk factors for cardiovascular disease, including hyperlipidemia, hypertension, obesity, and diabetes are related to inappropriate eating habits [17]. Increasing consumption of foods high in saturated fat with high calories and reducing consumption of complex carbohydrates, dietary fiber, fruits, and vegetables have a significant impact on controllable risk factors for cardiovascular disease [18]. Studies have shown that the diet of 73% of Iranians needs to change [19].

Health educators play an important role in training the population to adopt healthy behaviors and learn the risk factors and diseases [20]. To change behavior, health educators need to have a good understanding of the health and social characteristics, beliefs, attitudes, values, skills, and past behaviors of individuals [21]. Although the change of behavior is very challenging, considering a behavioral model will help to change one's beliefs about cardiovascular disease [22]. Evidence demonstrated several important factors that could predict and affect food behaviors in women such as knowledge, self-regulation, outcome expectations, outcome expectancies [23], attitude, subjective norms, PBC, and intention [24].

**Fig. 1:**
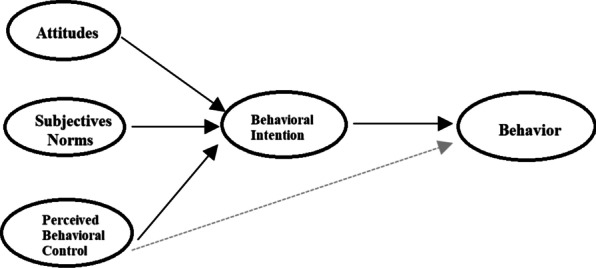
Theory of planned behavior (TPB)

TPB is a social-cognitive theory that provides a useful framework for predicting and understanding health-related behaviors [20, 25]. According to this theory, the intention is the primary determinant of behavior. The intention of the individual is influenced by the three factors of attitude, subjective norms, and PBC [26, 27] Conceptual framework of TPB is presented in Fig. 1.

The theory is one of the important models in food selection [28–30]. In a study by Emanuel et al., the application of the TPB to the consumption of fruits and vegetables was examined. The results showed that women had better-perceived attitudes and behaviors than men about fruits and vegetables, while men were more abstract [31]. Blanchard et al. investigated the application of the TPB to the consumption of fruits and vegetables 5 times a day among college students and examined that does gender or ethnicity affects this behavior. The results of the study showed that the TPB is a useful framework for intervening in the consumption of fruits and vegetables 5 times a day in men and women of different ethnicities and is not affected by gender and ethnicity [32]. Also, the results of the study by Gholami et al. showed that attitudes, habits, and tendencies have a direct impact on fruit and vegetable consumption. In general, the TPB can help predict and plan healthy behaviors and disease prevention [33]. A study by Hyun-sun Seo et al. investigated the application of TPB to the factors influencing fast food consumption among high school students in Seoul. The study showed that most students consume fast food under the influence of their friends as well as on special occasions and that girls are more interested in maintaining health and weight control than boys. The TPB indicated that fast food consumption was significantly influenced by behavioral tendencies [34]. Yarmohammadi et al. showed that the TPB provided a good predictor of the tendency to fast food consumption but is not very helpful in predicting behavior [35]. Rezabeigi Davarani et al. investigated the application of the TPB in predicting the factors affecting nutritional behaviors associated with cardiovascular disease among health volunteers in Kerman. This study showed that PBC is a strong predictor of food intentions and behaviors [24].

Consequently, considering the role of mothers in providing family food baskets and the effect of their nutritional behaviors on family members, the education of women can promote healthy eating behaviors in the community and family.

On the other hand, Given that understanding the factors affecting nutritional behavior and performing educational interventions will be of great help in preventing cardiovascular disease, especially among women, this study aimed to investigate the application of the TPB in nutritional behaviors related to cardiovascular diseases among the women in Fasa city, Fars province, Iran.

## Methods

### First stage study design

The study was conducted in two stages. First stage, the factors affecting nutritional behaviors associated with cardiovascular disease on 350 women who were referred to Fasa urban health centers were selected to determine the important factors affecting this behavior based on the TPB. This study was performed in January 2018- April 2019.

### First stage sample size and setting

This sample size was estimated based on Vahdani et al. [36] and Rezabeigi Davarani [24] with 350 individuals considering α = 5%, β = 20%, and sample loss.

$$n=\frac{{z}^{2}{s}^{2}}{{d}^{2}}=\frac{{(1.96)}^{2}\times {(0.47 )}^{2}}{({0.05)}^{2}}=\frac{(3.84)(0.226)}{0.0025}\cong 350$$

The study population consisted of women referred to Fasa health centers. After obtaining the necessary permits from Fasa University of Medical Sciences and Fasa Health Center, referring to selected centers (out of 6 Fasa Health Centers, 2 centers were randomly selected), availability sampling was performed from the women who visited the centers and had a family file. Then, by selecting each sample, the researcher introducing himself and stating the purpose of the study emphasized the confidentiality of the information. Finally, the questionnaires were completed by the patients. The inclusion criteria of the study were the willingness to participate, 20–50 years of age, and no pregnancy. Exclusion criteria included the unwillingness to participate and a history of cardiovascular disease.

### First stage statistical analyses

Data were analyzed by SPSS software with using logistic regression analysis. The significance level was considered 0.05.

### Second stage study design and setting

In the second stage, based on the results of a cross-sectional study, quasi-expeimental study was performed on 200 women covered by Fasa health centers in May 2019- September 2019. Out of 6 Fasa health centers, 2 centers were randomly selected (100 as the experimental group and 100 as the control group). Inclusion criteria included the willingness to participate, ages 20–50, and no pregnancy. Exclusion criteria included cardiovascular disease, unwillingness to continue the study, and absence for more than two educational sessions.

### Interventional program

The educational intervention for the experimental group consisted of eight 50–55 min’ sessions on the lecture, group discussion, question and answer, use of video and instructional images, and PowerPoint presentations. The educational program was performed by a Ph.D. expert in health education and health promotion with the collaboration of a nutritionist. The content of the educational sessions was the concepts related to the cardiovascular disease preventive diet. The details of the training sessions are presented in Table 1.

**Table 1:** The details of the training sessions

Sessions	Contents	Constructs	Presentations
First session	Introduction to cardiovascular disease and its symptoms, complications and diagnosis	Attitude	Lecture- group discussion- question and answer-use of video
Second session	A 55-year-old woman diagnosed with cardiovascular disease was invited as a model and talked to the subjects about cardiovascular diseases and its risk factors, symptoms, complications, and diagnosis with the help of a physician	Attitude-Subjective norms	Role playing- group discussion- use of video and instructional images-
Third and fourth sessions	The role of nutrition in preventing cardiovascular disease, benefits and barriers of diet, following dietary recommendations, perceived behavioral control in observing proper diet, and recording activities in the specified forms	Perceived behavioral control- Attitude-Nutritional performance	group discussion- question and answer-use of instructional images- PowerPoint
Fifth and sixth sessions	two sessions was held with the presence of at least one family member and the role of family members in making, facilitating, and providing suitable food was explained	Subjective norms-Intention	Lecture-group discussion- question and answer-PowerPoint
Seventh session	Nutritional behavior preventing cardiovascular disease -type and composition and amount of food, fruits and vegetables, etc Intention to consume proper nutrition	Intention- Nutritional performance- Perceived behavioral control	use of video and instructional images- PowerPoint
Eighth session	The previous sessions were reviewed and the subjects were provided with educational pamphlets	All constructs	Educational booklet- WhatsApp group

### Interventional procedure

Educational sessions were held for 4 groups of 25 patients (of experimental group) weekly in the health center. A WhatsApp group was formed to exchange information and send motivational and educational messages every 5 days. Two follow-up sessions were held 1 month later and 2 months after the intervention to review the material.

For ethical considerations, the study was approved by the Ethics Committee of Fasa University of Medical Sciences. Completing informed consent by the participants, the goals, importance, necessity of the study were discussed, and the participants were assured that their information would be treated completely confidential. Both experimental and control groups have participated from the beginning to the end of the study. The control group did not receive any educational program except that they were just invited to complete the questionnaire. To adhere to ethical standards, an educational session was held for the control group at the end of the study.

### Second stage study instrument

The tool used for the study was a questionnaire prepared based on former studies [24, 36–39]. The questionnaire consisted of 50 questions in 2 sections. The first section included demographic information (age, weight, height, household size, education, marital status, occupation, household income, family history of cardiovascular disease, history of tobacco use, and history of cardiovascular-related education). The second part included the constructs of the TPB such as attitude (positive and negative assessment of behavior), subjective norms, PBC, intention, and nutritional behavior that were designed and regulated by the researcher through the study of books, papers, and literature.

To measure attitude, subjective norms, and PBC, Likert scale ranging from 1 (strongly disagree) to 5 (strongly agree) was used: 10 items were used to measure attitude, for example, ‘I prefer to eat boiled food’ and ‘I think we should eat fruits and vegetables every day’; 6 items were used to measure subjective norms such as ‘My family members are interested that I cook foods according to the appropriate principles of nutrition’ and ‘Health workers encourage me to follow healthy diet’; 8 questions were asked to assess PBC, for example, ‘I can choose healthy food even if it doesn't taste delicious’ and ‘I can avoid the conditions that tempt me to eat unhealthy food; 7 behavioral intention questions were also scored on a five-point Likert scale ranging from very low to very high, such as ‘I intend to include at least 3 to 5 servings of vegetables and fruits a day’ and ‘I intend to incorporate fish 1 to 2 times a week into the diet’; and performance including 10 questions such as ‘I consume at least three fruits a day’ and ‘I put salt on the table when eating’ was measured on a Likert scale ranging from 1 to 5.

The validity of the items was evaluated by calculating the item impact score above 0.15 and the content validity ratio higher than 0.79. To determine the tool's face validity, a list of compiled items targeted by 30 women with similar demographic, economic, and social characteristics. To determine the content validity, the opinions of 12 experts (outside the research team) on health education and health promotion (n = 10), nutritionist (n = 1), and cardiologist (n = 1) were used. Using the Lawshe table index, the item which was 0.56 larger for 12 subjects, was considered essential and kept for further analysis. The overall reliability of the tool with Cronbach's alpha was 0.88. The construct validity of attitude was 0.84, PBC 0.82, abstract norms 0.89, behavioral intention 0.87, and performance 0.86. Also, Participants' height was measured using standing tape on the wall without shoes and weight with the minimum coverage and without shoes using digital scales. Finally, Body Mass Index (BMI) was calculated by SPSS software. Body mass index less than 18.5 was considered to be lean, 18.5–24.9 normal, 25.9–9.9 overweight, and 30 and above obese. The questionnaire was completed by the experimental and control groups before and three months after the intervention.

### Second stage study statistical analyses

Data of the second study were analyzed by SPSS 22 software The data in before analysis was the normal distribution. Demographic variables were compared between two groups with the Chi-square test. Comparison between the constructs of TPB and jogging performance was done with Paired t-test in groups. Constructs of TPB and jogging performance were also compared between two groups with an independent *t*-test. The level of significance is considered < 0.05.

## Findings

In the cross-sectional study, 350 women with the mean (± SD) age of 39.58 ± 4.86 years participated in the study. The results of the logistic regression analysis to predict nutritional behaviors related to cardiovascular disease based on the TPB are presented in Table 2. According to the results, all three constructs of attitude, subjective norms, and PBC were predictors of nutritional behaviors associated with cardiovascular disease in women. Generally, all the studied variables, predicted of the behavior (R^2^Adjusted = 0.28).

**Table 2:** Linear regression analysis of the factors related to nutritional behaviors associated with cardiovascular disease in women (N = 350)

Variables	Beta	STD β	*p*	Dependent variable
Attitude	0.179	0.111	0.034	Nutritional behaviors associated with cardiovascular disease R^2^ = 0.416 R^2^Adjusted = 0.28
Perceived behavioral control	0.272	0.071	0.014	
Subjective norms	0.182	0.102	0.018	

In the quasi-experimental study, the mean (± SD) age of women in the experimental group was 38.80 ± 4.75 and in the control group 39.25 ± 4.20 years (*p* = 0.194). Besides, the mean household size in the experimental group was 3.88 ± 2.68 and in the control group 3.74 ± 2.72 (*p* = 0.221), in which there was no significant difference between the two groups. The results of the interventional study indicated that the chi-square test showed a significant difference between the experimental and control groups in terms of occupation variables, household income, education level, marital status, BMI, family history of cardiovascular disease, and history of receiving cardiovascular education, and history of tobacco use (Table 3).

**Table 3:** Comparison of demographic variables of studied patients in two groups of experimental and control

Variables		Experimental group		Control group		P-value*
		Percentage	Number	Percentage	Number	
Marital status	Single	9	9	7	7	0.317
	Married	81	81	85	85	
	Divorced	4	4	5	5	
	Widowed	6	6	3	3	
Education level	Illiterate	1	1	0	0	0.262
	Primary school	7	7	6	6	
	Secondary school	28	28	32	32	
	High school	44	44	46	46	
	College	20	20	16	16	
Monthly income	Less than 20 million rials	42	42	37	37	0.178
	20–40 million rials	38	38	40	40	
	More than 40 million rials	20	20	23	23	
Smoking history	Yes	17	17	12	12	0.128
	No	83	83	88	88	
Family history of cardiovasculardiseases	Yes	22	22	18	18	0.106
	No	78	78	82	82	
Occupation	Housewife	71	71	64	64	0.218
	Employed	29	29	36	36	
History of educations related to cardiovascular diseases	Yes	14	14	12	12	0.246
	No	86	86	88	88	
BMI	Lean (less than 18.5)	4	4	3	3	0.209
	Natural (18.5–24.9)	52	52	49	49	
	Overweight (25–29.9)	35	35	38	38	
	Obese (more than 30)	10	10	9	9	

*The statistical tests used: The chi-square test

The results showed that there was no significant difference between the experimental and control groups regarding attitude, subjective norms, PBC, intention, nutritional performance related to the cardiovascular disease before the intervention, but three months after the intervention, compared to the control group, the experimental group showed a significant increase in the mentioned constructs (Table 4).

**Table 4:** Comparison of mean score of TPB constructs and nutritional performance in the experimental and control groups before and three months after the educational intervention

Variable	Group	Before intervention Mean ± SD	After intervention Mean ± SD	P-value*
Attitude	Experimental	24.32 ± 4.28	42.32 ± 4.03	< 0.001
	Control	25.12 ± 4.26	27.01 ± 4.11	0.122
	P-value**	0.216	*p* < 0.001	
Subjective norms	Experimental	14.20 ± 2.23	25.40 ± 2.56	< 0.001
	Control	14.87 ± 2.36	15.39 ± 2.35	0.316
	*p-value*	0.412	*p* < 0.001	
Perceived behavioral control	Experimental	18.10 ± 3.14	33.72 ± 3.47	< 0.001
	Control	17.79 ± 2.52	18.10 ± 2.41	0.323
	*p-value*	.337	*p* < 0.001	
Intention	Experimental	13.37 ± 2.25	30.13 ± 2.35	< 0.001
	Control	14.08 ± 2.16	15.14 ± 2.08	0.422
	*p-value*	0.289	*p* < 0.001	
Nutritional performance	Experimental	16.28 ± 3.56	41.38 ± 3.47	< 0.001
	Control	17.07 ± 3.64	18.15 ± 3.48	0.265
	*p-value*	0.269	*p* < 0.001	

*The statistical tests used: Paired t-test

**The statistical tests used: Independent sample t-test

## Discussion

An educational intervention to promote cardiovascular disease-preventing nutritional behaviors helps to disseminate information, change norms and social values related to cardiovascular risk factors, and awareness of the risk of the disease. The present study aimed to apply the TPB to nutritional behaviors associated with cardiovascular disease in women in Fasa city, Fars province, Iran. In this study, first, a cross-sectional study was conducted to determine the factors affecting nutritional behaviors related to cardiovascular disease in 350 women, whose results showed that attitude, subjective norms, and PBC predict nutritional behaviors related to cardiovascular disease in women. Also, 41.6% of the variance of behavior is explained by the constructs of the TPB. In a cross-sectional study conducted by Rezabeigi Davarani et al. on 128 women, attitudes, subjective norms, PBC, and intention predicted 37% of nutritional behaviors related to cardiovascular disease. PBC was the strongest predictor of intention [24]. The available evidence suggests that the application of the TPB for examining the power of predicting model in behavioral changes showed that, this model can predict around 20–30% of the observed variance of health behaviors. Although Ajzen demonstrated the effectiveness of TPB-based interventions in predicting the behavioral changes and found that attitude, subjective norm, and PBC accounted for a significant amount (20% to 78%) of variance in behavioral intention [40–42]. On the other hand, the systematic review study results showed that it is possible to facilitate behavior changes especially changes in nutrition with using the TPB [43].

In a qualitative study in which Sabzmakan et al. examined the experiences of patients with cardiovascular risk factors and health workers on the determinants of nutritional behavior, the main problem of the patients was that they were unable to follow a regular diet. The findings of this study showed that patients' nutritional behavior was affected by social support (subjective norms) [44].

In the White et al. study, participants (N = 184) completed questionnaires evaluating the standard TPB measures (attitude, subjective norm, and PBC) and additional voluntary planning to eating foods low in saturated fats. Self-report consumption of low unsaturated fats was assessed 1 month later. The results indicated that attitudes and subjective norms predicted intentions to eat foods low in saturated fats and intentions and PBC. Predicted low food intake in saturated fats directly as well as mediated intentional behavior and PBC of behavior, suggesting an important role for planning as a post-intentional construct determining healthy eating choices [45].

In Wu's study, the TPB predicted dietary sodium intake in heart failure patients [46]. In a study by Moshki and Torabi on lifestyle-related factors, based on the TPB, the attitude was a significant predictor of healthy eating intention [47]. In a study by Rahimi et al., Health Belief Model (HBM) constructs predicted 20% of the variance of intention to prevent cardiovascular disease behaviors [48].

In a study by Blanchard et al. on 215 cardiac patients, constructs of TPB predicted 30% of the variance of intention to exercise [49]. In Mullan et al.'s study, the TPB predicted 47.6% of changes in breakfast consumption [50]. Also, in the study of Yarmohammadi et al., the constructs of the theory were a poor predictor (6%) for fast food consumption behavior among the students in Isfahan [35]. In the study of Sassen et al., TPB constructs predicted 41% of the variance of intention to physical activity in patients with cardiovascular risk factors. Attitude, subjective norms, and PBC were the predictors of intention [51]. In a study by Moshki et al., HBM constructs predicted 17.3% of the variance of cardiovascular disease preventive behaviors [52].

The results of an interventional study indicated the effectiveness of an educational intervention based on the TPB in promoting nutritional behaviors associated with cardiovascular disease in women. The mean score of patients' attitudes toward dietary behaviors related to cardiovascular disease in the experimental group showed a significant increase in the three months after the intervention. Attitude is based on the consequences of one's own experience of behavior or succession experiences through observational learning from others [53]. For this reason, after experiencing a behavior directly, positive beliefs about the consequences of the behavior are reinforced and then as a motivator influence its continuity. Attitude also refers to feelings arising from behavior. The experience of these pleasant emotions can affect their promotion and continuation [54]. The favorable attitude of women towards healthy nutrition is probably due to the availability of educational texts, continuous attendance at educational sessions, the application of brainstorming, discussion on the consequences of healthy eating behavior by target group women, and experiencing positive physical and psychological benefits derived from it.

In a study by Ebrahimi et al., which examined the effect of educational intervention on promoting healthy eating behaviors among students, the results showed that educational intervention caused a positive and incremental change in the knowledge, attitude, and behavior of students in the experimental group on healthy eating [55]. In a clinical trial study conducted by Karimi et al. on 80 patients with myocardial infarction in Bandar Abbas, the experimental group participated in a 4-session educational intervention based on the TPB. The results showed that the attitude, subjective norms, and PBC increased significantly in the experimental group three months after the intervention [56]. The results of the study by Abbaszadeh et al. showed that educational intervention on patients with myocardial infarction improved their attitudes [57]. In the study of Cespedes et al., the educational intervention improved the knowledge, attitude, habits related to a healthy diet and a healthy lifestyle to prevent cardiovascular disease [58].

In the studies of Jeihooni et al. [59] and Shahmohammadi et al. [60], the educational intervention changed the positive attitude of participants' nutritional behaviors. The mean score of subjective norms showed a significant increase in the experimental group three months after the intervention in comparison with the control group. Holding an educational session using role-playing, group discussion, and question & answer (Q&A) with a cardiologist; and using their opinions, attending a family member in the educational session, and engaging health care staff in the training program as social supporters have an important role in improving the score of subjective norms and application of healthy diet behaviors related to cardiovascular disease. In a quasi-experimental study conducted by Salehi and Heydari on 118 women, the educational intervention was performed in three 45 min of lectures and Q&A sessions as well as a slide show for the experimental group. There was also a session with family members to discuss a healthy diet. Three months after the intervention, there was a significant increase in the mean scores of reinforcing factors (subjective norms) and attitudes toward cardiovascular disease-related diet behaviors [37]. The study of Azadbakht et al. showed that factors such as parents, friends, peers, and mass media influence dietary selection [61]. In Charkazi et al.'s study, mate's lack of encouragement and desire to use liquid oil was the most important barriers to appropriate nutritional performance [62]. In Robinson's study, family, friends, and peers played a key role in the nutritional behaviors of African Americans. Besides, the role of women was reported to be of an important emphasis on the interpersonal process that influences nutritional behaviors, as women are primarily responsible for supplying food and interested in improving their health habits [63].

The story also reports that the social environment including interaction with family, friends, and peers will influence dietary selections through mechanisms such as imitation patterns, social support, and social norms. Therefore, it is suggested that the individuals and families attend the educational classes, as family support can be effective in maintaining a diet [64]. In Kothe and Mullan's study, subjective norms related to fruit and vegetable consumption increased after the intervention [65]. In White et al. study, where the effect of educational intervention based on the TPB on promoting physical activity and healthy eating in elderly people with cardiovascular disease and type 2 diabetes were investigated, subjective norms of physical activity in the experimental group, compared to the control group, had a significant increase after the intervention, but there was no significant difference between the two groups in nutrition [66]. Research by Oil et al. highlighted the role of physicians and family support in the prevention of cardiovascular disease [67]. Doo et al. emphasized the role of the family and other subjective norms in nutritional behaviors [68]. In this study, the mean score of PBC in the experimental group was significantly higher than that of the control group in the three months after the intervention. Educating healthy eating behaviors, providing appropriate nutrition patterns through educational sessions, and providing appropriate encouraging and informative feedback in group discussions led women to an understanding of the benefits and barriers of appropriate nutritional behaviors preventing cardiovascular disease.

PBC means one’s perception of to what extent the behavior is under control. If the behavior is not completely under the control of the individual, even when he or she is strongly motivated by subjective norms and attitudes, the behavior may not be due to the interference of circumstances. If people believe that they do not have the resources to perform a behavior, they will probably not have a strong intention to do so. Even though they have a positive attitude toward the behavior or believe that others important to them also endorse the behavior, a sense of control will make them strive to succeed in what they want. As a result, they will adopt the behavior they intend to do [69]. In the study of Rezabeigi Davarani et al., lack of finances, dietary habits, taste, and odor are the most important barriers influencing healthy eating behavior [19]. Folta et al. cited major barriers to women's change in reducing cardiovascular risk factors, lack of support of different tastes, and cultural and economic factors [70]. In Moshki et al.'s study, a significant correlation was found between perceived barriers and cardiovascular disease preventive behaviors [52].

In a quasi-experimental study conducted by Didarloo et al., using the TPB to promote obesity preventive lifestyle, three months after educational intervention, mean score of attitude, subjective norms, PBC and the behavior of experimental group showed a significant increase [71]. Hosseini Soorand et al. investigated the effect of an educational program based on the TPB in patients with hypertension. After the intervention, the mean score of PBC, attitude, subjective norm, behavioral intention, and behavior in the experimental group showed a significant increase, but no significant difference was observed in the control group [72]. In a study conducted by Zainali et al. to investigate the effect of educational intervention based on the HBM on promoting preventive behaviors of cardiovascular diseases, after the intervention, the mean score of self-efficacy and preventive behaviors in the experimental group significantly increased and the perceived barriers decreased compared to the control group [38].

In a quasi-experimental study conducted by Rezabeigi Davarani et al. on 128 healthy volunteers to examine the effect of educational intervention based on the TPB on nutritional behaviors related to cardiovascular disease, the experimental group received 6 sessions of 90-min of educational intervention. Six weeks after the intervention, the mean score of knowledge, attitude, PBC, subjective norms, and nutritional behaviors increased significantly in the experimental group [73]. Other results were consistent with the findings of this study [74–76].

The results of this study showed that the mean score of intention and nutritional behaviors related to cardiovascular disease in the experimental group compared to the control group showed a significant increase after the intervention. According to the TPB, increasing the mean score of attitude, subjective norms, and PBC in the experimental group three months after the educational intervention increased their behavioral intention and also promoted nutritional behaviors preventing the cardiovascular disease, hence the positive impact of the educational program. The results of Bahadori Monfared et al.’s study showed that nutrition education and counseling are effective in improving nutritional behavior and correcting malnutrition behaviors [77].

In the Eqbali Ziyarat et al. study, MEDFICTS (Meats, Eggs, Dairy, Fried Foods, Baked Foods, Convenience Foods Table Fats, and Snacks Dietary Questionnaire) scores improved in patients with myocardial infarction receiving nutritional counseling. It seems that nutritional counseling surgery in patients with myocardial infarction may be effective in reducing the incidence of this disease [78]. The results of Kheiri et al.'s study showed that mean scores of the HBM constructs and CVD preventive behaviors were significantly increased in the experimental group compared to controls after the intervention [39].

In the interventional study of Zainali et al., 61 patients were included in the study and randomly divided into the intervention and control groups. The intervention group was educated for one month. Three months after the intervention, the mean score of awareness, self-efficacy, guidelines for practice, and preventive behaviors of cardiovascular disease was significantly increased in the intervention group compared to the control group. Also, perceived barriers in the intervention group were significantly reduced compared to the control group [38].

The results of Hassani et al.'s study showed significant differences between the knowledge scores and other items of the TPB model as well as nutritional indices observed in the intervention group after 3 months. Mean serum low-density lipoprotein (LDL) levels were significantly reduced after the intervention. Improvements in serum cholesterol (intragroup) and high-density lipoprotein cholesterol (HDL-C) (intergroup) levels were near the significant post-intervention group [79].

In the study of Karimi et al., the educational intervention based on the Theory of Planning Behavior increased the intention of patients with myocardial infarction (MI) to change their lifestyle in the experimental group [56]. In Wu et al.'s study, adherence to a low-salt diet in patients with heart failure, the results showed the effect of educational intervention based on the TPB on improving the nutrition of the experimental group [80]. In a study by Welsh et al., which examined the effect of educational intervention based on the TPB on low-salt diet among the patients with heart failure in 2012, the results showed that after 6 months, dietary sodium in the experimental group significantly decreased compared to the control group [81]. The results of other studies were consistent with the results of this study [82–84].

The present study had some limitations the most important of which was conducted only on women and the participants self-reported their nutritional behaviors which may raise the probability of recall and response bias. We decide to resolve it by underlining the confidentiality of during the data gathering process to participants. Also, the similarity of the approach of the present study in males or other groups of individuals is recommended for further studies.

## Conclusion

The results of the present study showed that the educational intervention based on the TPB increased the mean score of attitude, subjective norms, PBC, behavioral intention, and promoted the nutritional behaviors preventing cardiovascular disease in women. Considering the validity of the TPB for influence changing people’s beliefs and intentions toward nutrition behavior, therefore this model is recommended to apply and promote a healthy diet among patients with chronic diseases.

## Data Availability

The datasets used and/or analyzed during the current study can be made available by the corresponding author on reasonable request.

## Abbreviations

BMI: Body Mass Index
HBM: Health Belief Model
TPB: Theory of Planned Behavior
PBC: Perceived behavioral control
CVD: Cardiovascular disease
LDL: Low-density lipoprotein
HDL-C: High-density lipoprotein cholesterol
